# Constant Pressure-Regulated Microdroplet Polymerase Chain Reaction in Microfluid Chips: A Methodological Study

**DOI:** 10.3390/mi15010008

**Published:** 2023-12-20

**Authors:** Luyang Duanmu, Youji Shen, Ping Gong, Hao Zhang, Xiangkai Meng, Yuanhua Yu

**Affiliations:** 1 School of Physics, Changchun University of Science and Technology, Changchun 130022, China; duanmuluyang@163.com; 2 School of Life Science and Technology, Changchun University of Science and Technology, Changchun 130022, China; 17300067689@163.com (Y.S.); gp@cust.edu.cn (P.G.); zhanghao@cust.edu.cn (H.Z.); mxk2018@cust.edu.cn (X.M.)

**Keywords:** constant pressure regulation, microdroplet digital PCR, measurement of gas solubility, fluorescence detection, quantitative detection

## Abstract

Digital polymerase chain reaction (PCR) technology in microfluidic systems often results in bubble formation post-amplification, leading to microdroplet fragmentation and compromised detection accuracy. To solve this issue, this study introduces a method based on the constant pressure regulation of microdroplets during PCR within microfluidic chips. An ideal pressure reference value for continuous pressure control was produced by examining air solubility in water at various pressures and temperatures as well as modeling air saturation solubility against pressure for various temperature scenarios. Employing a high-efficiency constant pressure device facilitates precise modulation of the microfluidic chip’s inlet and outlet pressure. This ensures that air solubility remains unsaturated during PCR amplification, preventing bubble precipitation and maintaining microdroplet integrity. The device and chip were subsequently utilized for quantitative analysis of the human epidermal growth factor receptor (EGFR) exon 18 gene, with results indicating a strong linear relationship between detection signal and DNA concentration within a range of 10^1^–10^5^ copies/μL (R^2^ = 0.999). By thwarting bubble generation during PCR process, the constant pressure methodology enhances microdroplet stability and PCR efficiency, underscoring its significant potential for nucleic acid quantification and trace detection.

## 1. Introduction

In recent years, polymerase chain reaction (PCR) has gained prominence due to its extensive utility in genetic analysis and disease diagnosis [1,2,3]. Emerging from advancements in microfluidic platforms, digital PCR offers notable advantages over traditional quantitative real-time PCR (qPCR), such as reduced reaction volume, increased reaction speed, diminished system noise, and enhanced sensitivity [4]. Digital PCR methodologies can be broadly classified based on their droplet generation techniques. One approach involves partitioning nucleic acids into discrete micropores or microchambers [5], while the other entails diluting the target across a multitude of consistent microliter or nanoliter droplets [6]. Notably, droplet digital PCR has witnessed wider adoption, as it provides higher reaction volumes at a more economical cost [7]. This method involves dispersing the digital PCR reaction solution, which includes the test sample, across thousands of independent micro-reaction units. Post-PCR amplification, the fluorescence signals from each unit are interpreted [8]. The methodology involves calculating the number of negative and positive reactions, and leveraging statistical algorithms along with Poisson distribution software for results analysis, enabling the absolute quantification of target molecules [2,9]. This advanced technique has been utilized extensively in applications including but not limited to the study of circulating tumor DNA [10], copy number variation [11], gene expression [12], rare mutation detection, single cell analysis [13,14,15], and trace nucleic acid quantification [16,17].

Unfortunately, the existing classic instruments have the characteristics of a large volume, excessive heat loss, high power consumption, and a time-consuming and laborious nature, and they require more execution time. In addition, it is difficult to establish a digital PCR platform that integrates, automates, and miniaturizes micro devices on a separate testing and analysis platform [4]. One primary challenge within digital PCR technology lies in the generation of stable, abundant, and monodisperse droplets. These droplets must withstand critical conditions during PCR process, such as rapid temperature fluctuations and complex aqueous samples [18]. Added to this is the predicament of bubble generation and evaporation. The evaporation issue can be mitigated through the use of oil seals [19]. However, addressing bubble formation, ubiquitous in microfluidic systems and a significant reason for PCR failure on chips, demands further scrutiny [5]. 

During PCR process, bubbles manifest in two forms: inherent and newly formed. Inherent bubbles comprise those introduced during sample addition and those persisting within chip microstructure gaps. In contrast [20], newly formed bubbles primarily arise during PCR’s rapid heating and cooling process due to liquid degassing triggered by increased liquid temperatures [21]. These small bubbles act as vaporization nuclei, undergoing expansion as surrounding liquids continuously evaporate into them. As these bubbles reach saturation and the system temperature escalates, the vapor pressure inside these bubbles increases. Consequently, these growing bubbles migrate within the chip influenced by thermal convection, leading to large-scale droplet disruption. Currently, several researchers have acknowledged the detrimental effects of bubbles, leading to numerous studies on bubble removal [22]. Karlsson et al. employed a semi-permeable membrane to enable the escape of bubbles produced in the PCR chamber, while simultaneously preventing water loss. Jie et al. eliminated bubbles from liquid-filled microchannels by incorporating hydrophobic porous membranes at the microchannels’ apex [21]. In the study by Friedrich et al., gas bubbles created by degassing during thermocycling were expelled through capillary-driven transport within tapered regions of the PCR chamber [23].

Karlsson et al. have noted that prior mechanisms for bubble removal in microsystems were reliant on membranes, porous structures, and capillary action. However, these methods may inadvertently extract a significant volume of liquid along with the gas, and they ensure the PCR process by eliminating bubbles. Among these techniques, the fabrication of chips can be challenging, or the microsystems may be complex and not readily scalable for industrialization. These limitations underscore the necessity for an integrated, cost-effective ddPCR system that can reliably expel bubbles from shallow cavities and is user-friendly. In this paper, we introduce a method for constant pressure regulation of droplet PCR in microfluidic chips. This approach can inhibit the formation of new bubbles and the precipitation and movement of inherent bubbles during the PCR process, thereby ensuring the stability of microdroplets. In addition, the robustness of this constant pressure regulatory device, when integrated with the chip, is verified through nucleic acid quantitative tests targeting the epidermal growth factor receptor (EGFR) gene. This gene holds potential as a molecular indicator for early DNA mutation detection. Identifying these mutations paves the way for early tumor detection and provides guidance for molecular-targeted therapies [24]. Therefore, the research on microdroplet PCR in microfluidic chips, based on constant pressure regulation, holds great significance. It not only serves as a beacon for refining droplet-based digital PCR technology but also has broader ramifications in the realm of medical diagnostics and therapeutic guidance.

## 2. Materials and Methods

### 2.1. Reagents and Instruments

Reagents and instruments included droplet generation oil for probes (1863005, BIO-RAD, Hercules, CA, USA), forward primers (YUANQI-BIO, Shanghai, China), reverse primers (YUANQI-BIO), MGB probes (YUANQI-BIO), pGEM plasmids embedded in EGFR exon gene 18 sequence (YUANQI-BIO), ddPCRSupermix for probes (BIO-RAD), ddPCR gene chip reader (Changchun Jite, Changchun, China), ddPCR gene chip (Changchun Jite, Changchun, China), Manta g-1236 CMOS camera, 200 μL centrifuge tube (AXYGEN, Hangzhou, China), micropipette (Eppendorf, Hamburg-Nord, Germany), suction head (AXYGEN), handheld centrifuge (Eppendorf), pressure calibrator (DPI611-07G), temperature calibrator (FLUKE, Everett, WA, USA), Peltier (ETX6-12-F1-3030-TA-RT-W6), flexible trachea (SMC), aluminum plate, PT1000, TM115 temperature control module (Chengdu Yexian, Chengdu, China), 42 stepper motor, sealed through hole rubber plug, barometer (BD-1001XB), screw rod, heating rod (CT0670), PT100, sealing piston, measuring bottle, seal plate, stirring paddle, and pressure sensor (MPX5050GP), as well as solenoid valve (Parker). ImageJ v4.4 (National Institutes of Health, Bethesda, MD, USA) software is adopted to process images captured in experiments.

### 2.2. Increasing Pressure to Rise Gas Saturation Solubility

Gases are present as dissolved substances and microbubbles within the droplet solution of the ddPCR chip, influenced by Henry’s Law and Fick’s Law of Diffusion. These laws dictate that lowering the system temperature or elevating the pressure can increase gas solubility, leading to enhanced dissolution of microbubbles in the droplet solution. However, during the PCR process, the temperature peaks at 98 °C, which diminishes the solubility of gases, resulting in the precipitation of gases from the solution and the formation of microbubbles that may disrupt the droplets. Thus, maintaining a temperature during the droplet PCR process that does not compromise solubility is crucial to prevent bubble formation. Investigating the effects of temperature and pressure on gas solubility is essential. For this purpose, we have developed a solubility measurement apparatus and employed the static saturation method to ascertain gas solubility at varying temperatures and pressures.

### 2.3. Constant Pressure Control Device and ddPCR Gene Chip

**Figure 1:**
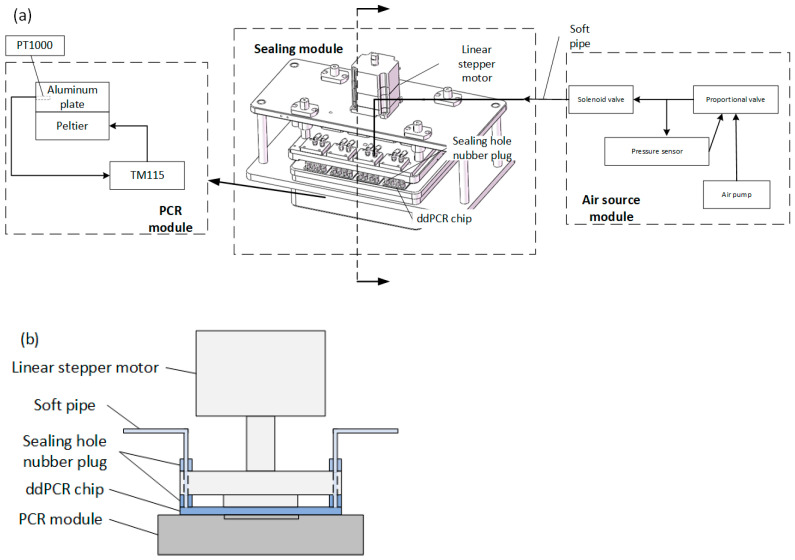
Schematic diagram of constant pressure control device (**a**); section diagram of sealing device (**b**).

The experimental device includes three primary modules: PCR, sealing, and gas source modules, as shown in Figure 1a. The cross-sectional diagram of the sealing device is shown in Figure 1b.

PCR module: tailored for rapid temperature changes during amplification of nucleic acid samples, this module is bifurcated into a heating and a temperature control subset. The former is composed of two Parcel patches arrayed adjacently under an aluminum plate and serially connected to a power source. Conversely, the temperature control subset employs TM115 module of Chengdu Yexian Technology, PT1000 is a platinum thermistor, and its resistance value changes with temperature. This module combines with PT1000 to collect the temperature of the aluminum plates. Leveraging proportional integral (PID) closed-loop regulation, it facilitates brisk temperature escalation and reduction, achieving an aluminum plate surface temperature accuracy of ±0.3 °C and a uniformity of ±0.3 °C.

Sealing module: this segment orchestrates the compression of the sealing pressure plate to encapsulate the microfluidic chip. Integral components encompass a sealing pressure plate, a linear stepper motor, and two sealing through-hole rubber plugs. As the sealing pressure plate is descended, the rubber plug tightly attached to the chip’s inlet and outlet, while fixing the chip, ensuring hermetic integrity during droplet generation and PCR phase.

Gas source module: designed to provide stable pressure for microdroplet formation and PCR, this module primarily includes an air pump, pressure sensor, proportional valve, and a solenoid valve. Utilizing PID closed-loop regulation, it consistently produces stable air pressure with a mere fluctuation margin of ±0.1 millibar during droplet creation. An ancillary chip fixture is used to fix the droplet generation chip, equipped with a potentiometer to modulate the air pressure. Under a continuous operational span of 2 h, the apparatus manifests a fluctuation range of ±1 millibar.

**Figure 2:**
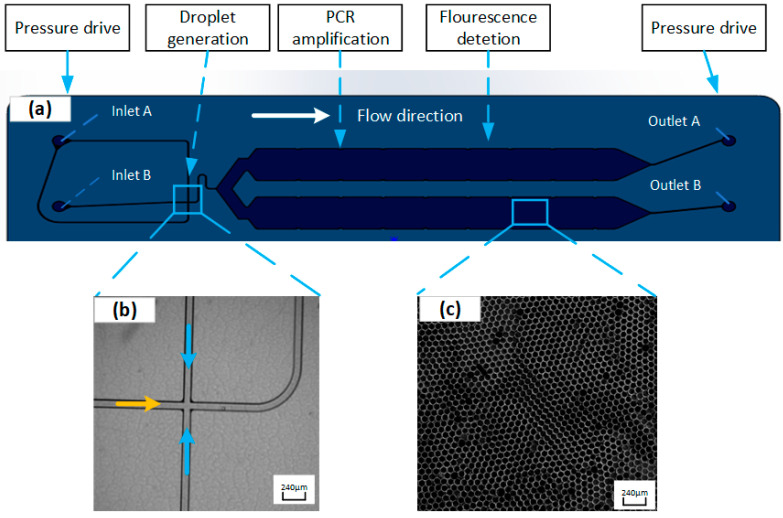
Schematic diagram of the integrated droplet digital polymerase chain reaction (ddPCR) gene chip structure (**a**). Flow focus structure of droplets generation part (blue arrows represent the flow direction of the oil; orange arrow represents the flow direction of sample) (**b**). Single layer arrangement of droplets in the collection chamber (**c**) (color figure online).

**Figure 3:**
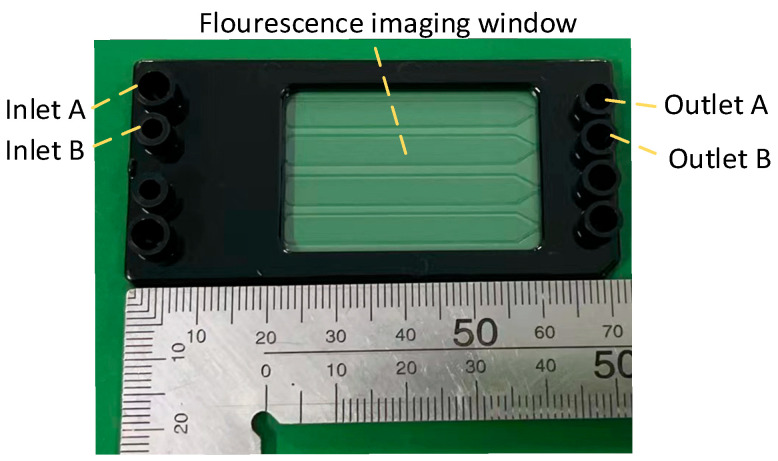
Image of the chip.

The ddPCR chip used in this study is shown in Figure 2 and Figure 3. Samples and oil were introduced into the left inlet of the chip and were driven by pressure into the droplet generation region where droplets were formed. The droplets then entered the microchannels of the chip. PCR amplification was performed in the microchannels of the chip under pressure, followed by imaging of the droplets.

### 2.4. Data Acquisition and Analysis

Upon the conclusion of PCR amplification, the PCR transfers the chip to a gene chip image reader for observation. Stimulated by 485 nm (FAM, a carboxyl fluorescence probe corresponding to a 485 nm laser) excitation, the fluorescence microscope imaging system and CMOS image sensor are employed to take photos for capturing the fluorescence images of PCR reaction results. Moreover, ImageJ software is applied to count the number of positive droplets and total droplets. According to Poisson’s statistical principle, the absolute amount of target DNA contained in the respective reaction can be calculated using Equation (1) and the practical number of positive droplets [25]:

(1) c=−n×ln⁡1−dnVd×n

where c is the concentration of target DNA in the sample, n is the total number of droplets in the chip, d is the number of positive droplets, and $V_{d}$ is the volume of each droplet.

## 3. Experimental Results and Discussion

### 3.1. Derivation of Gas Saturation Solubility

**Figure 4:**
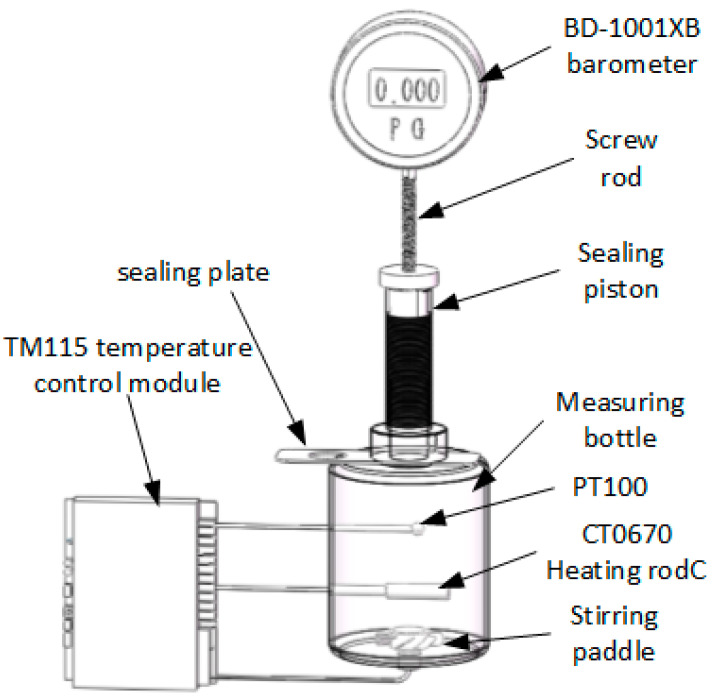
Gas solubility measurement device.

As depicted in Figure 4, the experimental apparatus comprises two primary modules: the temperature control module and the pressure control module. These modules facilitate the determination of air solubility in water across different temperatures and pressures.

The temperature control module encompasses key components such as TM115 temperature control meter, CT0670 heating rod, and PT100 temperature sensor. Utilizing a PID closed-loop control system, this module ensures accurate temperature control of the liquid in the measuring bottle can be achieved. The core components of the pressure control module of the liquid within the measuring bottle.

On the other hand, the pressure gauge module integrates core components like a BD-1001XB pressure gauge, a screw mechanism, and a sealing piston. The primary function of this module is to control the vertical motion of the sealing piston, enabling efficient pressurization.

**Figure 5:**
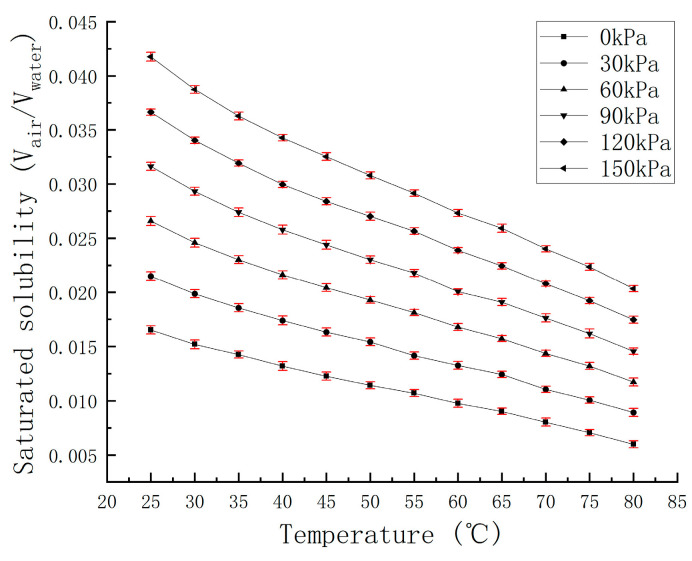
The solubility curve of air under different pressures as a function of temperature.

The saturation solubility of air in water was measured through experiments, and the solubility curve with temperature under constant pressure was fitted, curve as shown in Figure 5.

**Figure 6:**
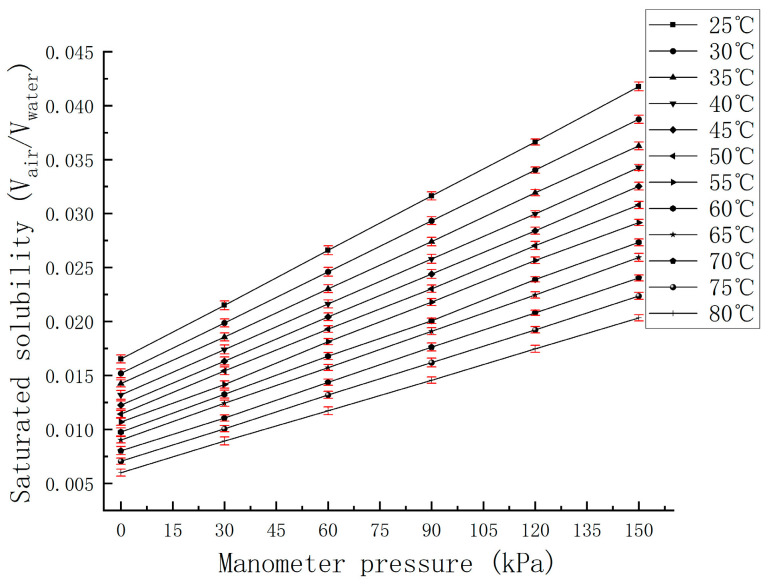
The solubility curve of air at different temperatures as a function of pressure.

**Figure 7:**
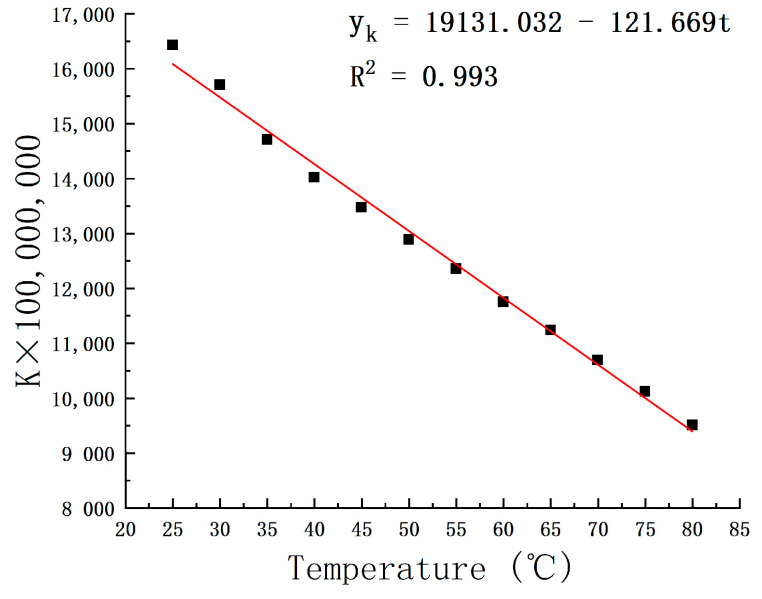
K × 100,000,000-value curve with temperature variation.

**Figure 8:**
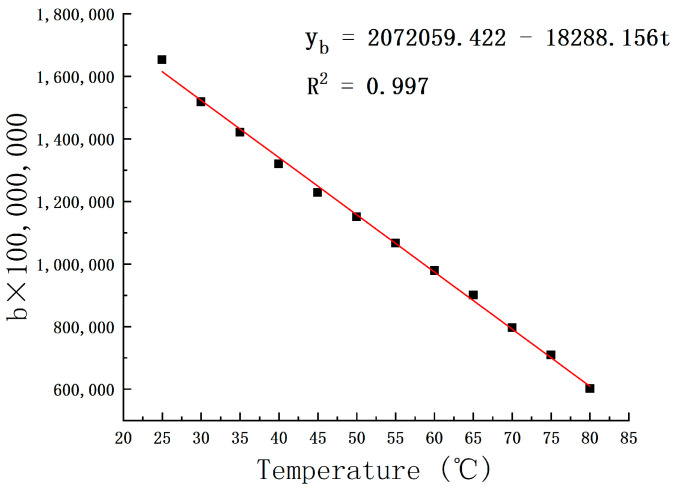
b × 100,000,000-value curve with temperature.

The above data were transposed to obtain the solubility curve with pressure at a constant temperature, transpose curve as shown in Figure 6:

Then, the relationship between air solubility and pressure at constant temperature can be obtained. The slope K and intercept b are shown in Table 1 below:

Curve fitting of slope data and intercept data is shown in Figure 7 and Figure 8:

From the analyses undertaken, a deduced relation emerges: when the temperature is denoted as T and the pressure value as P, the solubility S can be represented by the equation S=K×P+b. The K×100,000,000 formula is:

(2) $$\begin{array}{c} y_{K}=19131.032-121.669t \end{array}$$

The b×100,000,000 formula is:

(3) $$\begin{array}{c} y_{b}=2072059.422-18288.156t \end{array}$$

where $y_{K}$ is K×100,000,000, $y_{b}$ is  b×100,000,000, and *t* is temperature.

Considering the variance in environmental temperatures across regions, an initial temperature of 35 °C has been designated. At this temperature, water’s saturation solubility stands at 0.0143. By incorporating the experiment equation,  t=98 °C and P=150 kPa, S=0.0147>0.0143. This represents that at 98 °C, a system pressure exceeding 150 kPa ensures the absence of bubble formation within the chip.

### 3.2. Mitigating Bubble Formation during PCR Process

Based on the research results of the preliminary experiments, a series of test pressure values, namely 800 mbar, 1150 mbar, 1500 mbar, and 2000 mbar, were established. The pressure of the device was meticulously calibrated using a specialized pressure calibrator. Observational results indicate that over an interval of 15 min, the average pressure maintained was approximately 1150.4 mbar, experiencing minimal fluctuations of about ±0.2 mbar. Subsequently to this calibration, the chip, previously filled with the microdroplets as mentioned, was positioned in the device, and PCR spanning 40 temperature cycles was initiated. It is paramount to underscore that throughout this PCR process, specific temperatures—50 °C, 72 °C, 95 °C, and 98 °C—were assigned to measure temporal temperature changes on the aluminum plate’s surface. Data analysis indicated that the deviation between the actual surface temperature of the aluminum plate and the predetermined temperature is confined to a margin of 0.5 °C. Furthermore, when assessing the temperature consistency across six random locations on the aluminum plate’s surface, the variance was found to be within a range of 0.5 °C, repeat measurements three times per group.

**Figure 9:**
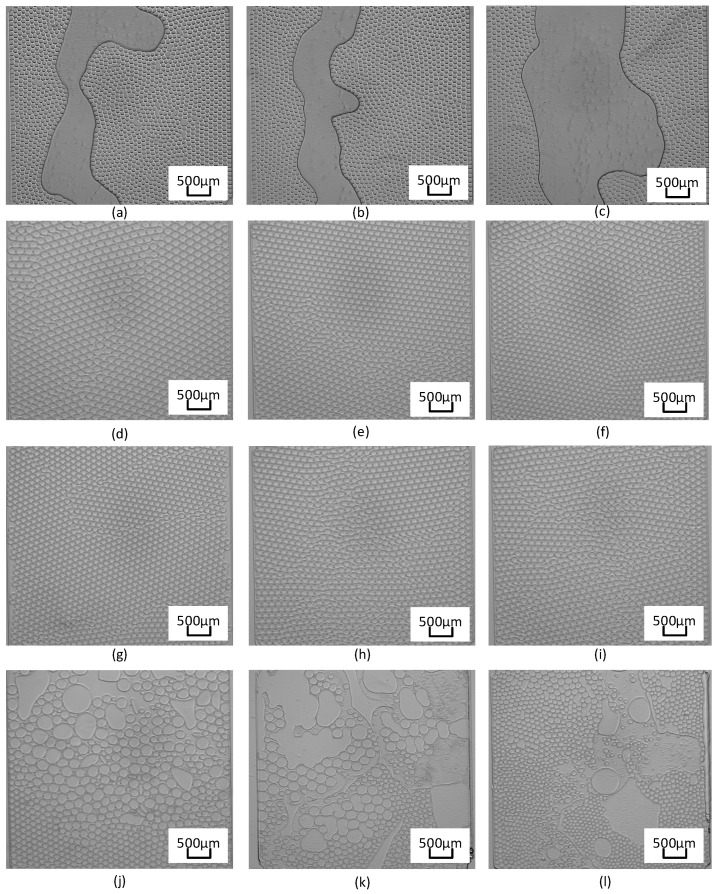
Droplet PCR results under different pressures. (**a**–**c**) PCR result at 800 mbar, (**d**–**f**) PCR result at 1150 mbar, (**g**–**i**) PCR result at 1500 mbar, and (**j**–**l**) PCR result at 2000 mbar.

During PCR process, as the temperature rises, the pressure gradually escalates to 1150 mbar. After peaking at 1150 mbar, the pressure continued throughout the remainder of PCR, eventually descending slowly to atmospheric levels. At the same time, set 800 mbar, 1500 mbar, and 2000 mbar pressure for measurement. Depressurization results are shown in Figure 9. An analysis of these results reveals the following: at a pressure of 800 mbar, the majority of microdroplets within the chip deteriorate, leading to the large bubble, as illustrated in Figure 9a–c; at 1150 mbar pressure, the chip’s microdroplets remain undamaged, as shown in Figure 9d–f; at 1500 mbar pressure, the chip’s microdroplets remain undamaged, as shown in Figure 9g–i; for the 2000 mbar pressure setting, an observed phenomenon includes the coalescence of droplets within the chip, as evidenced in Figure 9j–l. Upon comparing the outcomes at 800 mbar and 2000 mbar pressures, droplet degradation is evident in both scenarios, albeit stemming from disparate causes. Specifically, at 800 mbar pressure, due to the re-precipitation of gas in the solution, bubbles are formed, and after the bubbles fuse and become larger, they move and gather inside the chip to form giant bubbles that run through the chip; conversely, under the influence of 2000 mbar pressure, excessive intracavitary pressure instigates shear forces, precipitating droplet fusion. At this time, this condition does not result in new gas desorption or the genesis of large bubbles, the volume of undamaged droplets is compressed. Compared with the pressure of 1250 mbar and 1500 mbar, the droplet inside the chip is intact, although the droplet can maintain a normal form under the condition of rapid temperature change. The pressure of 1150 mbar is precisely at the minimum critical pressure that can ensure that the droplets are not damaged, and the pressure of 1500 mbar is what we consider reasonable. At the same time, the pressure obtained under a pressure of 2000 mbar is beyond the reasonable pressure range. Microdroplet diameters at 1150 mbar pressures are about 88.3 μm, 85.2 μm, and 84.6 μm, while microdroplet diameters at 1500 mbar pressures are approximately 80.3 μm, 81.8 um, and 79.8 μm.

### 3.3. Fluorescence Detection

**Figure 10:**
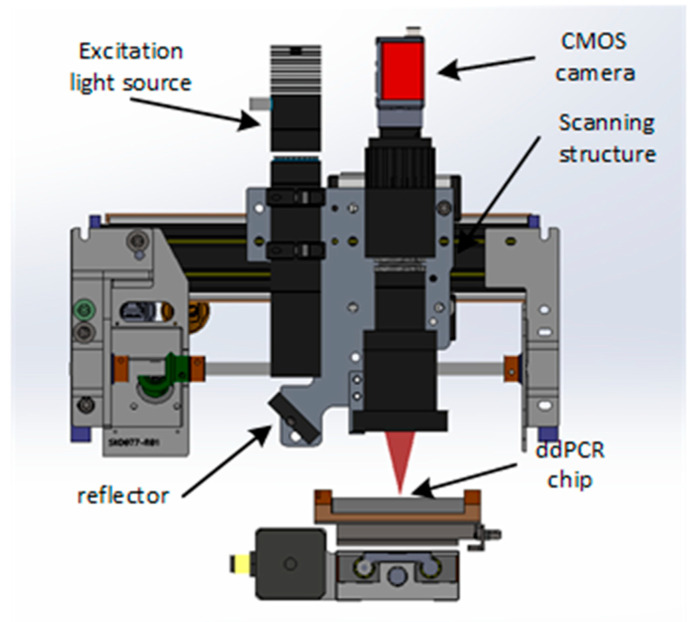
Fluorescence detection device.

**Figure 11:**
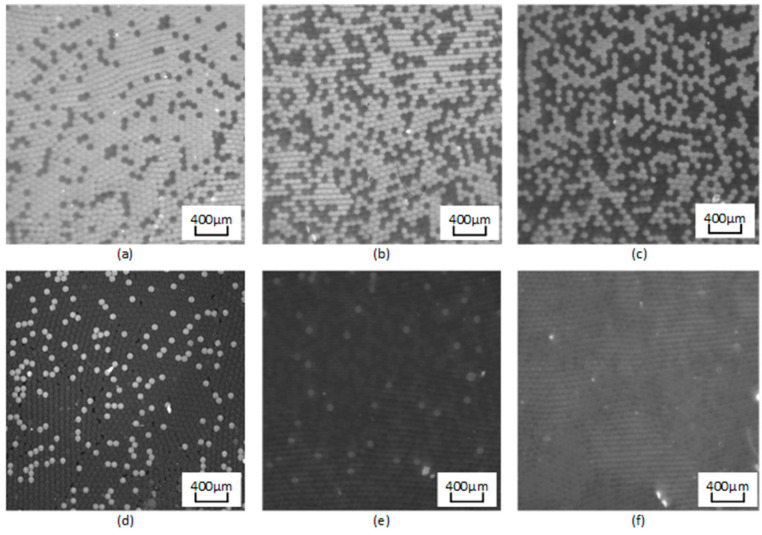
Fluorescence images for simultaneous detection of epidermal growth factor receptor (EGFR) exon 18 gene with various DNA concentrations. DNA concentration (copies/μL): (**a**) 10^5^; (**b**) 10^4^; (**c**) 10^3^; (**d**) 10^2^; (**e**) 10^1^; (**f**) negative control.

Following PCR amplification, the chip was transferred to ddPCR gene chip reading instrument for fluorescence signal measurement, with outcomes displayed in Figure 10. The probe’s interaction with the target gene prompts the fluorescent reporter’s release. As a result, droplets containing the template exhibit a pronounced fluorescent signal when exposed to excitation light. In contrast, droplets without the template show only a weak background fluorescence. The disparity in fluorescence intensity serves as a metric to designate droplets as either positive or negative. Central to ddPCR technology is the identification and quantification of negative/positive droplets. ImageJ v4.4 software (National Institutes of Health) is adept at gauging the gray values of droplets in fluorescence images and distinguishing fluorescence intensities to set a fluorescence threshold. Specifically, droplets with fluorescence signals exceeding the threshold are classified as positive, while those beneath are deemed negative. According to Poisson statistical principle, the study corroborated nucleic acid’s quantitative detection, leveraging ddPCR gene chip constant pressure regulation technique with EGFR exon 18 gene as the focal DNA molecule. Additionally, a dilution process of pGEM plasmid solution containing EGFR exon 18 gene yielded a five-tier concentration gradient standard ranging from 10^1^ copies/μL to 10^5^ copies/μL. As shown in Figure 11a, with a sample concentration of 10^5^ copies/μL, positive droplets near saturation. However, as the template concentration dwindles from 10^5^ copies/μL to 10^1^ copies/μL, there’s a commensurate decline in positive droplets, as portrayed in Figure 11b–e.

**Figure 12:**
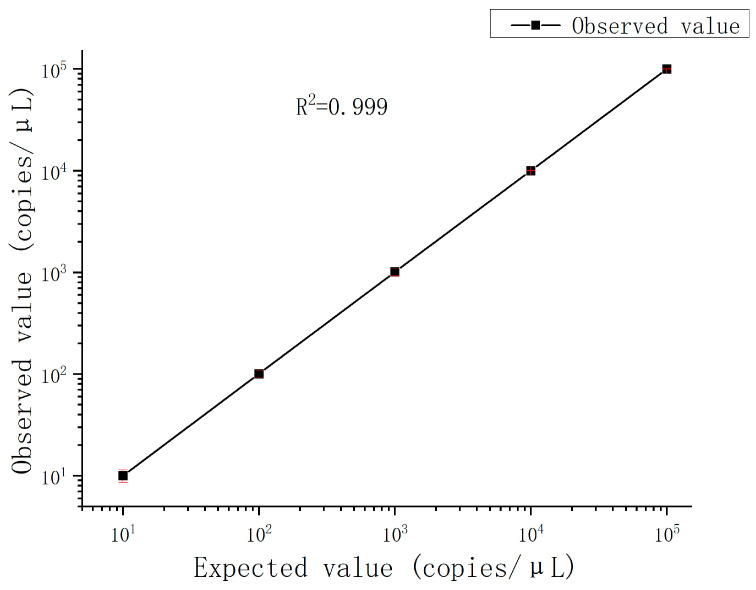
Observed value in the integrated droplet digital polymerase chain reaction (ddPCR) gene chip against expected value. The error bars represent the 95% confidence interval.

The fluorescence images are captured after 40 thermal cycles, and the fluorescence signal is determined via the fluorescence excitation channel of EGFR exon 18 gene with light excitation at 485 nm (FAM). In accordance with Poisson’s statistical principle, Equation (1) is adopted to calculate the absolute amount of target DNA in the respective reaction system. The concentration of DNA templates is calculated based on the results of fluorescence detection systems for samples with different concentration gradients. Subsequently, the standard curve is generated (Figure 12). After the result of ddPCR gene chip reading in detecting the target gene concentration of 1 × 10^1^ to 1 × 10^5^ copies/μL is summarized, the results reveal a good linear relationship.

## 4. Conclusions

The device was developed for droplet PCR within a microfluidic chip, utilizing constant pressure regulation. It effectively mitigates droplet instability during PCR amplification, which is typically induced by bubble formation, while also ensuring droplet stability throughout the PCR cycle, thus enhancing subsequent fluorescence detection. A thorough examination of the digital PCR process revealed that gas precipitation, prompted by fluctuations in solution temperature, is the primary cause of bubble emergence. We empirically ascertained the saturated solubility of water across a pressure range of 0 to 150 kPa and temperatures from 25 °C to 80 °C, applying linear regression to the data obtained. The findings indicate that maintaining a minimum pressure of 115 kPa at both ends of the microfluidic chip prevents gas precipitation within the solution. A series of droplet PCR experiments were performed on the microfluidic chip, setting pressures at 800 mbar, 1150 mbar, 1500 mbar, and 2000 mbar. The results demonstrated that at pressures of 800 mbar and 2000 mbar, droplets disintegrated; conversely, at pressures of 1150 mbar and 1500 mbar, the droplets remained stable without bubble formation. Further analysis of these outcomes was conducted to elucidate the variations in droplet integrity under different pressures. Concurrently, it was observed that the droplet diameter under a pressure of 1500 mbar was marginally reduced compared to that at 1150 mbar. Quantitative detection of EGFR exon 18 gene fragment was undertaken using a ddPCR gene chip reader. There emerged a robust linear correlation when DNA concentrations ranged between 10^1^ copies/μL and 10^5^ copies/μL. Therefore, the microdroplet PCR approach within microfluidic chips, based on constant pressure regulation, boasts significant efficacy. This method not only ensures droplet PCR integrity on the microfluidic chip but also retains the precision of fluorescence detection and subsequent analysis.

## Figures and Tables

**Table 1:** Slope K and intercept b.

Temperature (℃)	K	b	K × 100,000,000	b × 100,000,000
25	0.000164286	0.016528571	16,428.57143	1,652,857.143
30	0.000157048	0.01518254	15,704.7619	1,518,253.968
35	0.000147111	0.014211111	14,711.11111	1,421,111.111
40	0.00014019	0.013196825	14,019.04762	1,319,682.54
45	0.000134762	0.012287302	13,476.19048	1,228,730.159
50	0.000128921	0.011503175	12,892.06349	1,150,317.46
55	0.000123619	0.010661905	12,361.90476	1,066,190.476
60	0.000117524	0.009785714	11,752.38095	978,571.4286
65	0.000112413	0.009007937	11,241.26984	900,793.6508
70	0.000106952	0.007961905	10,695.2381	796,190.4762
75	0.000101238	0.007090476	10,123.80952	709,047.619
80	0.000095142	0.006014286	9514.285714	601,428.5714

## Data Availability

The data that support the findings of this study are available from the corresponding author upon reasonable request.

## References

[1] Auroux P.A., Koc Y., Demello A., Manz A., Day P.J.R. (2004). Miniaturised nucleic acid analysis. Lab A Chip.

[2] Gingeras T.R., Russell H., Kricka L.J., Dennis L.Y., Wittwer C.T. (2005). Fifty years of molecular (DNA/RNA) diagnostics. Clin. Chem..

[3] Roper M.G., Easley C.J., Landers J.P. (2005). Advances in polymerase chain reaction on microfluidic chips. Anal. Chem..

[4] Kulkarni M.B., Goel S. (2020). Advances in continuous-flow based microfluidic PCR devices—A review. Eng. Res. Express.

[5] Liu H.B., Gong H.Q., Ramalingam N., Jiang Y., Dai C.C., Hui K.M. (2007). Micro air bubble formation and its control during polymerase chain reaction (PCR) in polydimethylsiloxane (PDMS) microreactors. J. Micromech. Microeng..

[6] Meng X., Yu Y., Gong P., Jin G. (2021). An integrated droplet digital PCR gene chip for absolute quantification of nucleic acid. Microfluid. Nanofluid..

[7] Hou Y., Chen S., Zheng Y., Zheng X., Lin J.-M. (2022). Droplet-based digital PCR (ddPCR) and its applications. TrAC Trends Anal. Chem..

[8] Goel S. (2021). Microelectronics and Signal Processing: Advanced Concepts and Applications.

[9] Wei C., Yu C., Li S., Meng J., Li T., Cheng J., Li J. (2022). A droplet-based multivolume microfluidic device for digital polymerase chain reaction. Sens. Actuators B Chem..

[10] Diaz L.A., Bardelli A. (2014). Liquid biopsies: Genotyping circulating tumor DNA. J. Clin. Oncol..

[11] Whale A.S., Huggett J.F., Simon C., Valerie S., Jacqui S., Stephen E., Foy C.A., Scott D.J. (2012). Comparison of microfluidic digital PCR and conventional quantitative PCR for measuring copy number variation. Nucleic Acids Res..

[12] Hashimoto-Torii K., Torii M., Fujimoto M., Nakai A., El Fatimy R., Mezger V., Ju M.J., Ishii S., Chao S.H., Brennand K.J. (2014). Roles of heat shock factor 1 in neuronal response to fetal environmental risks and its relevance to brain disorders. Neuron.

[13] Zhong Q., Bhattacharya S., Kotsopoulos S., Olson J., Larson J.W. (2011). Multiplex digital PCR: Breaking the one target per color barrier of quantitative PCR. Lab A Chip.

[14] Mu D., Yan L., Tang H., Liao Y. (2015). A sensitive and accurate quantification method for the detection of hepatitis B virus covalently closed circular DNA by the application of a droplet digital polymerase chain reaction amplification system. Biotechnol. Lett..

[15] Chen L., Zhang C., Yadav V., Wong A., Senapati S., Chang H.-C. (2023). A home-made pipette droplet microfluidics rapid prototyping and training kit for digital PCR, microorganism/cell encapsulation and controlled microgel synthesis. Sci. Rep..

[16] Zhang H., Jenkins G., Zou Y., Zhu Z., Yang C.J. (2012). Massively parallel single-molecule and single-cell emulsion reverse transcription polymerase chain reaction using agarose droplet microfluidics. Anal. Chem..

[17] Kulkarni M.B., Goel S. (2023). Mini-thermal platform integrated with microfluidic device with on-site detection for real-time DNA amplification. BioTechniques.

[18] Dawson S.J., Tsui D.W., Murtaza M., Biggs H., Rueda O.M., Chin S.F., Dunning M.J., Gale D., Forshew T., Mahler-Araujo B. (2013). Analysis of circulating tumor DNA to monitor metastatic breast cancer. N. Engl. J. Med..

[19] Yin K., Zeng X., Liu W., Xue Y., Li X. (2019). Stable Colloidosomes Formed by Self-Assembly of Colloidal Surfactant for Highly Robust Digital PCR. Anal. Chem..

[20] Bian X., Jing F., Li G., Fan X., Jia C. (2015). A microfluidic droplet digital PCR for simultaneous detection of pathogenic *Escherichia coli* O157 and Listeria monocytogenes. Biosens. Bioelectron..

[21] Xu J., Vaillant R., Attinger D. (2010). Use of a porous membrane for gas bubble removal in microfluidic channels: Physical mechanisms and design criteria. Microfluid. Nanofluid..

[22] Karlsson J.M., Haraldsson T., Laakso S., Virtanen A., Mäki M., Ronan G., Van Der Wijngaart W. PCR on a PDMS-based microchip with integrated bubble removal. Proceedings of the 2011 16th International Solid-State Sensors, Actuators & Microsystems Conference.

[23] Schuler F., Trotter M., Geltman M., Schwemmer F., Wadle S., Domínguez-Garrido E., López M., Cervera-Acedo C., Santibáñez P., von Stetten F. (2015). Digital droplet PCR on disk. Lab Chip.

[24] Nakayama T., Hiep H.M., Furui S., Yonezawa Y., Saito M., Takamura Y., Tamiya E. (2010). An optimal design method for preventing air bubbles in high-temperature microfluidic devices. Anal. Bioanal. Chem..

[25] Wang P., Jing F., Li G., Wu Z., Cheng Z., Zhang J., Zhang H., Jia C., Jin Q., Mao H. (2015). Absolute quantification of lung cancer related microRNA by droplet digital PCR. Biosens. Bioelectron..

